# Dual contrastive learning based image-to-image translation of unstained skin tissue into virtually stained H&E images

**DOI:** 10.1038/s41598-024-52833-7

**Published:** 2024-01-28

**Authors:** Muhammad Zeeshan Asaf, Babar Rao, Muhammad Usman Akram, Sajid Gul Khawaja, Samavia Khan, Thu Minh Truong, Palveen Sekhon, Irfan J. Khan, Muhammad Shahmir Abbasi

**Affiliations:** 1 Department of Computer and Software Engineering, National University of Sciences and Technology, Islamabad, Pakistan; 2 Center for Dermatology, Rutgers Robert Wood Johnson Medical School, Somerset, NJ 08873 USA; 3 Department of Dermatology, Weill Cornell Medicine, New York, NY 10021 USA; 4 Department of Pathology, Immunology and Laboratory Medicine, New Jersey Medical School, 185 South Orange Ave, Newark, NJ 07103 USA; 5 EIV Diagnostics, Fresno, CA USA; 6 Department of Pathology, St. Luke’s University Health Network, Bethlehem, PA 18015 USA; 7 Department of Internal Medicine, Greater Baltimore Medical Center, Towson, MD 21204 USA; 8 University of California, San Francisco School of Medicine, San Francisco, USA

**Keywords:** Computational biology and bioinformatics, Medical research, Engineering

## Abstract

Staining is a crucial step in histopathology that prepares tissue sections for microscopic examination. Hematoxylin and eosin (H&E) staining, also known as basic or routine staining, is used in 80% of histopathology slides worldwide. To enhance the histopathology workflow, recent research has focused on integrating generative artificial intelligence and deep learning models. These models have the potential to improve staining accuracy, reduce staining time, and minimize the use of hazardous chemicals, making histopathology a safer and more efficient field. In this study, we introduce a novel three-stage, dual contrastive learning-based, image-to-image generative (DCLGAN) model for virtually applying an "H&E stain" to unstained skin tissue images. The proposed model utilizes a unique learning setting comprising two pairs of generators and discriminators. By employing contrastive learning, our model maximizes the mutual information between traditional H&E-stained and virtually stained H&E patches. Our dataset consists of pairs of unstained and H&E-stained images, scanned with a brightfield microscope at 20 × magnification, providing a comprehensive set of training and testing images for evaluating the efficacy of our proposed model. Two metrics, Fréchet Inception Distance (FID) and Kernel Inception Distance (KID), were used to quantitatively evaluate virtual stained slides. Our analysis revealed that the average FID score between virtually stained and H&E-stained images (80.47) was considerably lower than that between unstained and virtually stained slides (342.01), and unstained and H&E stained (320.4) indicating a similarity virtual and H&E stains. Similarly, the mean KID score between H&E stained and virtually stained images (0.022) was significantly lower than the mean KID score between unstained and H&E stained (0.28) or unstained and virtually stained (0.31) images. In addition, a group of experienced dermatopathologists evaluated traditional and virtually stained images and demonstrated an average agreement of 78.8% and 90.2% for paired and single virtual stained image evaluations, respectively. Our study demonstrates that the proposed three-stage dual contrastive learning-based image-to-image generative model is effective in generating virtual stained images, as indicated by quantified parameters and grader evaluations. In addition, our findings suggest that GAN models have the potential to replace traditional H&E staining, which can reduce both time and environmental impact. This study highlights the promise of virtual staining as a viable alternative to traditional staining techniques in histopathology.

## Introduction

Histopathology involves the microscopic examination of tissues to detect and diagnose diseases. Conventionally, tissue samples are processed by formalin fixation, paraffin embedding, tissue slicing, and staining for histopathological analysis^1,2^. Staining enhances the visualization of cellular features, enabling the identification and differentiation of pathological features^1^. The most widely used stain is the hematoxylin and eosin (H&E) stain, which accounts for 80% of all staining performed globally^3^. H&E staining involves the use of hematoxylin, a basic dye that complexes with nucleic acids or other negatively charged molecules, typically staining them blue-purple, and eosin, an acidic dye that stains membranes and most proteins pink^1,2,4^.

**Figure 1:**
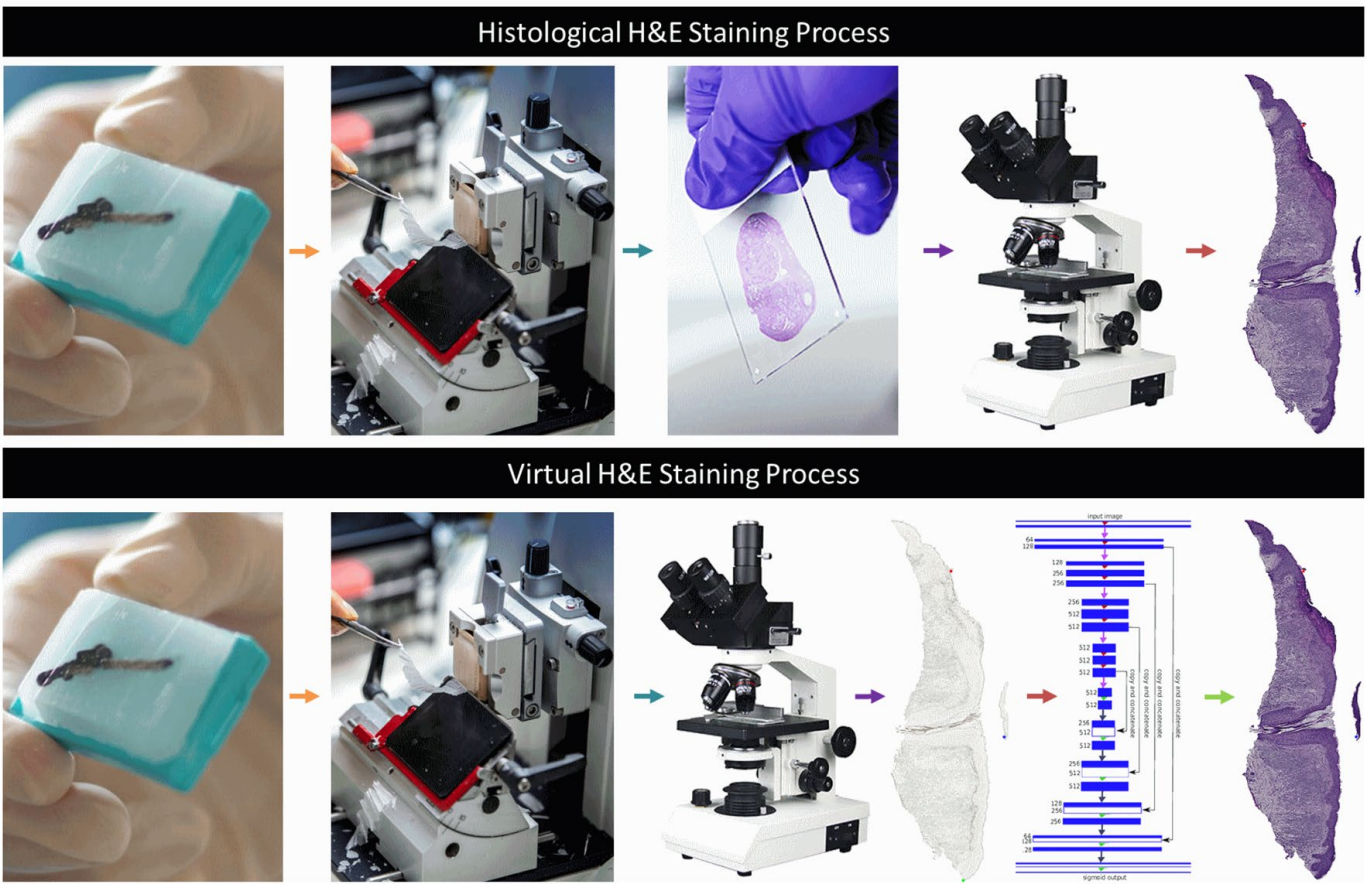
Traditional staining workflow (Upper) vs Virtual Staining workflow (Below). The chemical process of staining has been replaced with deep learning-based virtual staining.

There are two common workflows for tissue preparation and H&E staining. The primary workflow involves fixing tissues in formalin and embedding them in paraffin blocks. The blocks are then cut into thin sections (2–5 microns), hydrated, mounted on glass slides, deparaffinized, and stained for viewing under a brightfield microscope. This staining method takes approximately two hours^5^. The rapid staining workflow, on the other hand, is used primarily by surgeons in the operating room to obtain rapid diagnostic information, such as assessing tissues and measuring tumor margins. In this process, tissues are placed in a block of optimal cutting temperature compound instead of paraffin and then stained. This workflow takes 10–20 min, but the staining quality is generally lower than the standard laboratory approaches. The lower quality is caused by artifacts introduced during rapid freezing, which can deteriorate cell morphology and decrease diagnostic accuracy^2,5^. To address the limitations of traditional H&E staining, various optical diagnostic methods have been developed that aim to enhance tissue contrast without the need for fixation, embedding, and other chemical treatments. These techniques include nonlinear, confocal, photoacoustic, fluorescence, and multi-photon microscopes^2,5,6^. These methods produce black-and-white or intensity-based pseudo-colored images that differ from standard H&E stains and some techniques like Reflectance Confocal Microscopy and Optical Coherence Tomography can be costly and require trained operators and readers^2^. AI virtual staining offers a promising alternative for processing unstained samples that is cheaper, faster, and more consistent than traditional staining^2–7^. It reduces the time, labor and cost of tissue staining, as it does not require any specialized equipment, reagents, or personnel. One of its advantages is that it preserves the actual tissue, whereas traditional staining is destructive in nature and most of the time the stained tissue sections cannot be used for further analysis. Another advantage is that it avoids the use of many chemicals common in traditional staining that are harmful, known irritants, or toxic such as formalin, ethanol, xylene, and hematoxylin. Furthermore, deep learning based virtual staining provides results more quickly in mere minutes, compared to the traditional staining process that being a manual process can take up to several hours. Therefore, Virtual staining offers several benefits over the conventional method. Figure 1 compares the process of histological staining with virtual staining.

Generative Adversarial Networks (GANs) have been used in various ways to improve histopathology imaging, such as normalizing inconsistent tissue coloring^8^, converting H&E-stained images to special stains^3^, and detecting pathological features in tissues with virtual staining^9^. GANs have also been used to convert images from alternate microscopy techniques to their equivalent bright field H&E-stained images^2,10^. GANs are a type of neural network that can be thought of as two agents in a zero-sum game. The generator network captures the data distribution from the ground truth data and generates new examples, while the discriminator network classifies between ground truth and generated data. During training, both networks compete to improve their performance: the generator tries to create more realistic examples, while the discriminator tries to better identify false ones. When training is complete, the GAN model converges to a point where the discriminator can no longer differentiate between real and generated data, meaning that the generator has successfully captured the data distribution and produces examples that are indistinguishable from real data. There are different variations of GANs, such as Cycle GAN^11^, pix2pix^12^, and pix2pixHD^13^ which use different techniques to improve the results and reduce the model complexity.

In this study, a dual contrastive learning GAN model is used to translate unstained tissue slides into virtually generated H&E-stained slides. Dual Contrastive Learning GAN (DCLGAN)^14^ is an unsupervised image-to-image translation model that builds on the strengths of CycleGAN^11^ and Contrastive Unpaired Translation (CUT)^15^. The original CycleGAN model uses cycle consistency to train the model, which involves calculating the difference between the original image of domain A and the image obtained after transforming domain A image to domain B and then back to domain A. Another variation of this concept is learning between two image domains from an intermediate, shared content latent space, examples are Unsupervised Image-to-Image Translation Networks (UNIT)^16^ and Multimodal Unsupervised Image-to-Image Translation (MUNIT)^17^. This restricting approach forces the original and reconstructed image to be identical, making it hard to perform in cases where significant geometrical transformations are involved between image domains. In contrast, DCLGAN^14^ uses contrastive learning to maximize mutual information between input and generated images. Contrastive learning first introduced in CUT^15^ aims to learn feature embeddings where linked and related features are brought closer to each other in contrast to other samples in the dataset. This straightforward approach of maximizing mutual information by learning a cross domain similarity function generates better results, when compared with cycle consistency that aims to maintain similarity in content but not in appearance. DCLGAN^14^ improves CUT^15^ by extending learning one side mapping to learning two sided mappings across the domains. This idea of learning two sided mapping is borrowed from Cycle GAN^11^, and improves the performance in learning embeddings, achieving better results for image to image translation.

## Related works

Research in similar areas has shown that Generative Adversarial Networks (GANs) can be used for a range of pathological tasks, including stain normalization, virtual staining, tissue segmentation, and stain transformation from H&E to special stains. Runz et al.^8^ used a Cycle GAN (cycle consistent Generative Adversarial Network)^11^ for color normalization of H&E stains. They utilized Mitos-Atypia-14 challenge dataset and H&E Staining variation dataset for model training. The H&E stain normalized images obtained from Camelyon16 and Tumor lymph node datasets were used to train tumor classifiers to evaluate whether there are improvements from stain normalization. Results showed that Fréchet Inception distance improved by up to 96%. As different kind of dye staining of biopsy tissues is the standard criteria of cancer diagnosis, Rana and his team from MIT^7^ used deep learning to computationally generate stain prostate core biopsy H&E images. The study used 46 biopsy samples of 38 men having different Gleason graded prostatic adenocarcinoma. 102 scanned pairs of slide images were registered to generate 87,000 paired RGB 1024 × 1024 sized square patches. A conditional generative adversarial neural network (cGAN) model was trained on this data so that it could virtually generate H&E-stained images and another model was trained to de-stain H&E images back to their native non stained state. Computationally stained H&E images were compared with H&E-stained images by five pathologists revealing that 95% annotations were same in both cases. Kernel activation maps, generated from various model layers, were used to explain and interpret the underlying model. Pradhan et al.^18^ used Non-linear multimodal (NLM) imaging, which combined coherent anti-Stokes Raman scattering, two-photon excitation fluorescence and second-harmonic generation as input to computationally stain NLM images to H&E stain using the supervised methodology of conditional generative adversarial networks by using pix2pix^12^ model and unsupervised methodology using cycle generative adversarial networks (Cycle GANs)^11,18^. The training dataset consisted of tissue samples collected in another study from biopsies of patients with Crohn’s disease obtained through colonoscopy or surgical resections. The results were statistically significant and promising, prompting the need for further studies.

A supervised stain transformation network was trained by Haan et al.^3^ on spatially registered kidney tissue image pairs. Different models were trained to transform H&E stains to various special stains. The goal was to evaluate several non-neoplastic kidney diseases. To achieve subpixel spatial image registration, the input images for the actual style transformation network were generated using a CycleGAN network. The H&E stains were transformed to virtually generated Masson’s Trichrome stain, jones silver stain and Periodic acid–schiff stain. The results were presented to 3 pathologists with a memory washout period of three weeks. Statistical improvement in diagnosis was observed and confirmed through a t-tailed test in the 58 cases that were evaluated. Rivenson et al.^19^ proposed Phase Stain, a staining methodology that used a generative adversarial network to transform the label-free quantitative phase images (QPI) of tissue into brightfield microscopy images, which were similar looking to those stained with histological dyes. The team mapped QPI of human skin, kidney, and liver tissues to H&E, Jones stain, and Masson’s trichrome stain from paired image data. The results demonstrated that their method could create images that resembled histological stains in quality and appearance. Yang et al.^20^ used a cascaded deep neural network (C-DNN) for generating virtual stains. The C-DNN was trained on kidney needle core biopsy tissue sections in two steps: first, a deep neural network is trained to virtually stain label-free autofluorescence images into H&E stain. Second, another deep neural network transfers the H&E stained images into Periodic acid–Schiff (PAS) stain. Li et al.^9^ used deep learning for virtual histological staining of bright-field microscopic images of unlabeled carotid artery tissues. The analysis was used to find features such as media, intima, collagen, and elastic lamina. The adopted approach focused on using GAN for staining carotid tissue samples. The dataset consisted of artery samples collected from euthanized rats. Sixty whole slide image scanner (WSI) images each for unstained, H&E, orcein and PSR stains were collected for a total of 240 images. A cGAN that used U-Net^21^ and PatchGAN^22^ based architecture was trained to map unstained images to virtually stained images. Another GAN model STARGAN,^23^ that can learn multiple stain mappings simultaneously was used to generate H&E, PSR and Orcein stains from a single unstained tissue sample. Three pathologists graded the virtual stains on their quality and were able to reliably identify pathological features with a high degree of agreement between chemically stained and virtually stained images.

This work highlights that there continues to be a dependence on histopathology, especially in the surgical field. Biopsy specimens may be insufficient to undergo multiple staining techniques required for diagnosis^1^. While efforts have been made to use virtual staining and improve it by defining quality control tests^24^, most of the research work is focused on improving alternative microscopy techniques. Virtual staining for brightfield microscope images, particularly the application of virtual staining for stain-to-stain transformation or transforming unstained tissue images to H&E-stained images is still a novel research area. There is a lack of publicly available datasets for training virtual staining models, as unstained tissues are rarely stored for diagnostic analysis. Aligning stained and unstained images is another issue that arises due to the physical deformations in tissues during staining. Additionally, the appearance of stained tissue can vary depending on factors such as the type of tissue, staining solutions, and duration of the staining process. These variations can occur across different labs and even within batches processed at different times at the same lab. Therefore, new algorithms and techniques are required to improve the accuracy and reliability of virtual staining. Traditional H&E stain requires the use of chemicals including xylene, ethanol, hydrochloric acid, alcohol, ethanol, hematoxylin, & eosin, paraffin wax, which are corrosives, irritants, and/or are flammable^25^. Virtual staining reduces this safety concern. In addition, virtual staining builds a secure and durable histopathology database that can be used for further artificial intelligence studies as compared to traditional H&E slides which may fade.

## Material and methods

### Dataset

**Figure 2:**
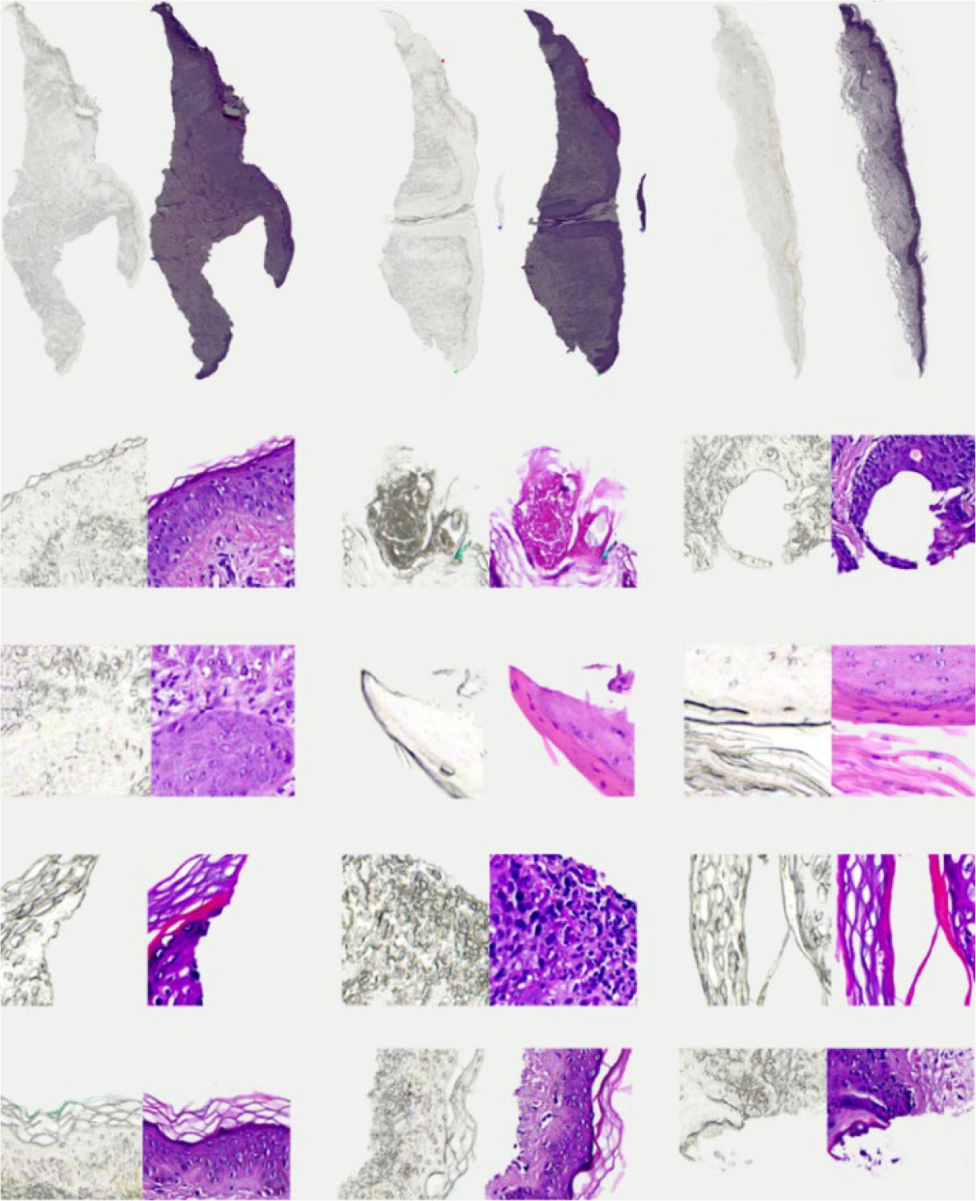
Skin tissue dataset. Row 1 shows three whole slide tissue sample pairs (unstained and stained with H&E). Rows 2–5 show patch pairs (unstained and stained with H&E).

The dataset used to train the virtual staining model consisted of 56 pairs of unstained and H&E-stained skin tissue slides. Most of these microscopic slides had more than one tissue placed on it and the total number of tissues pairs when saved separately in individual files returned 92 matching tissue pairs of unstained and H&E stained tissue. The images were obtained from a dermatology clinic and were de-identified. The images were scanned at a 20 × magnification using a standard brightfield microscope. Each image was divided into 512 × 512-pixel patches with a 256 pixel overlap between consecutive patches. Using the available data more than 40,000 paired patches from unstained and H&E-stained images were generated. However, a significant portion of these patches primarily consisted of plain background therefore they were excluded to ensure the data quality and relevance. After this refinement process, a total of 13,824 usable patches were obtained. Out of these, 11,000 patches were allocated for model training, providing a substantial dataset for the model to learn from. The remaining 2824 patches were used for testing, allowing us to assess the model's performance on unseen data. This partitioning results in a balanced training and testing data split of nearly 80:20. Figure 2 shows some randomly selected whole slide and small patch tissue images in the form of unstained and stained pairs.

Data augmentation in model training was not used, this decision was taken to follow the common pathology practice, where pathologists review tissues in specific orientations to ensure accuracy and standardization. The usual process of tissue examination involves aligning upper tissue layers e.g. Epidermis and its sublayers at the top and placing deeper layers e.g. subcutaneous tissue (Hypodermis), and deeper layers at the bottom. During data collection, experimental protocols were approved by the Advarra Institutional Review Board. All research methods were carried out in accordance with the relevant guidelines and regulations. Patients were informed about the research procedures, demonstrated competence, and provided informed consent.

### Methodology

**Figure 3:**
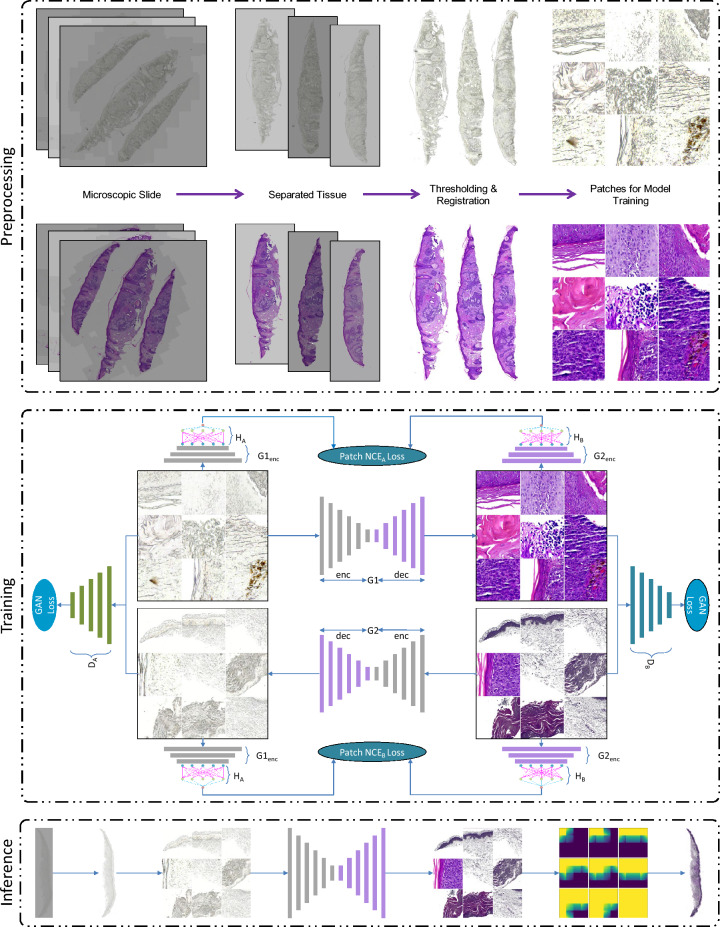
Overview of the proposed virtual H&E staining workflow showing preprocessing, training, and inference stages.

The proposed deep learning-based workflow (Fig. 3) for transforming unstained tissue samples into virtually generated H&E-stained images consists of three main stages: preprocessing, training, and inference. First, tissue samples are preprocessed to obtain single samples and remove background using adaptive thresholding. Due to potential misalignment between unstained and H&E-stained tissues during digital capture, registration is then performed to spatially align the images. Next, the tissue images are divided into smaller patches for both model training and testing. During the training stage, pairs of unstained and H&E-stained patches are used to train the models. The trained models are then applied in the inference stage to generate virtual H&E-stained patches from unstained patches. Finally, these patches are merged using alpha blending to create the complete virtually stained whole slide tissue sample.

### Preprocessing

Images of unstained tissue samples were obtained using a brightfield microscope, and the tissues were stained using the standardized laboratory approach for H&E staining. However, spatial misalignment often occurred due to difficulties in placing the tissue slides at the exact same position under the microscope or the presence of staining artifacts. Therefore, it was necessary to register both slides to align paired images for GAN-based image translation^26,27^. To achieve this, we used Scale Invariant Feature Transformation (SIFT), a feature detection algorithm that is invariant to rotation, depth, illumination, and scale. SIFT was used to identify key points from both images, and matching key points were then used to calculate the Homography matrix, which was applied to the unstained tissue image to register it with the stained tissue image. The resulting registered pair of images was then divided into 512 × 512 patches for model training and any patches that were blank or only consisted of a background were removed from the dataset. This registered dataset was then used to train our proposed model to learn the nonlinear data mapping between unstained and stained tissues.

### Model training (dual contrastive learning GAN (DCLGAN))

**Figure 4:**
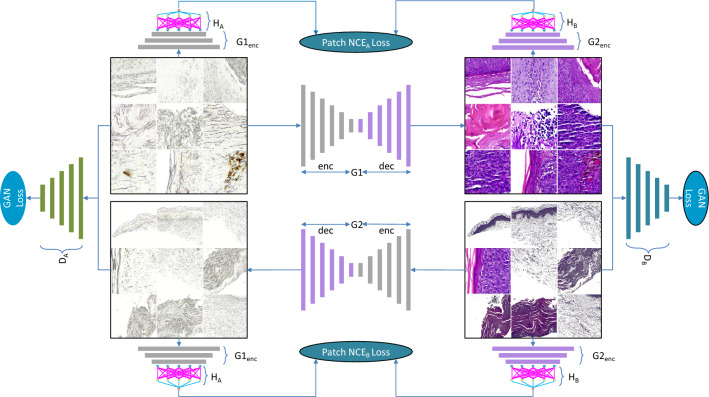
DCLGAN architecture involves learning two mappings: G1: A → B and G2: B → A. The encoded half of G1 and G2 is then labeled as G1enc and G2enc, respectively. G1enc and H_A_ serve as the embedding for A, while G2enc and H_B_ serve as the embedding for B^14^.

DCLGAN^14^ uses contrastive learning to maximize the mutual information between input and generated image patches. Contrastive learning, first introduced in CUTGAN^15^ aims to learn feature embeddings where linked and related features are brought close to each other in contrast to other samples in the dataset. This straightforward approach of maximizing mutual information by learning a cross-domain similarity function, instead of using cycle consistency. This aims to maintain similarity in content but not in appearance. DCLGAN^14^ improves CUT^15^ by extending learning one-sided mapping to learning two-sided mappings across the domains. This idea of learning two-sided mapping is borrowed from CycleGAN^11^ and improves the performance in learning embeddings, achieving better results in image-to-image translation tasks. The motivation for the DCLGAN topology as shown in Fig. 4 is to maximize mutual information by learning the correspondence between input and output image patches using separate embeddings. By using different encoders and projection heads for different domains, the model learns suitable embeddings to maximize mutual information. The dual learning setting also helps in stabilizing model training. DCLGAN consists of four main components: two generators (G_A_ and G_B_), two discriminators (D_A_ and D_B_), and two multilayered perceptron (H_A_ and H_B_) having two layers each to get feature embeddings. The generators are responsible for mapping images from one domain to another, such as from unstained to H&E stained. The discriminators are responsible for distinguishing between real and fake images in each domain, such as between real and generated images. The two multilayered perceptron modules are responsible for learning the correspondence between input and output image patches using separate embeddings. As DCLGAN employs distinct encoders and projection heads for each domain, this allows it to better learn domain-specific representations compared to CUT and effectively bridge the domain gap. This is crucial for accurate image translation. The dual learning setting where both forward and backward image-to-image translations are performed also helps to stabilize the training process and reach convergence.

For two given image domains $\textcircled{A}$ $\subset {\mathbb{R}}^{H\times W\times C}$, and $\textcircled{B}$ $\subset {\mathbb{R}}^{H\times W\times 3}$, where the H, W and C represent the height, width, and channel respectively, the dataset A consisting of set of images a, dataset B consisting of set of images b, belonging to domains $\textcircled{A}$ and $\textcircled{B}$ respectively, are denoted as $A = \left\{a\in \textcircled{A}\right\}$ and $B = \left\{b\in \textcircled{B}\right\}$.

Figure 4 shows the proposed DCLGAN architecture for skin tissue image virtual staining. It consists of two pairs of generators (G1, G2) and discriminators (${{\text{D}}}_{{\text{A}}}$, ${{\text{D}}}_{{\text{B}}}$). Generator G1 is trained for the mappings between domain A to B and G2 learns the inverse mapping from B to A. The discriminators make sure that the images belong to the correct domain while learning to map. Generators are made of encoders ${G1}_{\left(enc\right)}, {G2}_{\left(enc\right)}$ and decoders ${G1}_{\left(dec\right)}, {G2}_{\left(dec\right)}$. Features from image patches are passed from encoder ${G1}_{\left(enc\right)}, {G2}_{\left(enc\right)}$ to two multilayered perceptron (MLP) ${H}_{A}$ and ${H}_{B}$ having two layers each. The projected features learned by ${G1}_{\left(enc\right)} \& {H}_{A}$ are the embeddings used for training $G1: A \to B$ and similarly ${G2}_{\left(enc\right)} \& {H}_{B}$ are the embeddings used for training $G2 : B \to A$.

#### Mutual information maximization

**Figure 5:**
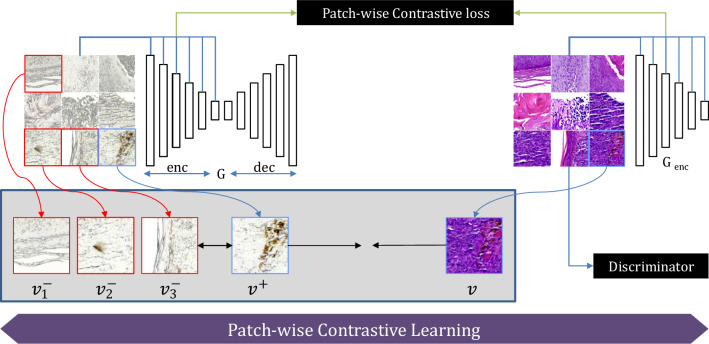
Patchwise contrastive learning^15^ maximizes mutual information between input and output patches, enabling one-sided translation in unpaired settings. This is done using a multilayer patchwise contrastive loss that maximizes mutual information between corresponding input and output patches and its corresponding input patch (positive example ${v}^{+}$) over other random patches (negative example ${v}^{-}$).

The objective of contrastive learning is to maximize the mutual information between input and output image patches^14,15,28^. A patch showing an unstained skin tissue feature with a particular identifying texture should associate closely and more strongly with a similar stained tissue feature having a similar texture. The generated images should look like the input used to generate it instead of resembling random images from the dataset. Contrastive learning is applied by using a Noise Contrastive Estimation (NCE) framework. The concept behind contrastive learning is to associate the two given image patches, The first image patch is called "query", and it is taken from the generated output, and it is compared with the input image patch taken from the same location, this second image patch is called a positive example. The negative image patches are taken from the same image but from different coordinate positions from the positive example, where $N$ is the number of negative patches (Fig. 5).

Query, positive, and negatives patches are mapped as $K$ dimensional vectors and are represented with $v,{v}^{+}\in {R}^{K}$ and ${v}^{-}\in {R}^{N\times K}$ respectively. ${v}_{n}^{-}\in {R}^{K}$ indicates $nth$ negative value. This can be simplified as $\left(N+1\right)$ classification problem which is done after normalizing vectors using L2 normalization. The likelihood of selecting the positive example against negatives is calculated and represented mathematically as cross entropy loss.

$$\mathcal{l}\left(v,{v}^{+},{v}^{-}\right)=-log\left[\frac{{\text{exp}}\left(sim\left(v,{v}^{+}\right)/\tau \right)}{{\text{exp}}\left(\mathit{sim}\left(v,{v}^{+}\right)/\tau \right)+\sum_{n=1}^{N}{\text{exp}}\left(\mathit{sim}\left(v,{v}_{n}^{-}\right)/\tau \right)}\right]$$

The expression $\mathit{sim}\left(u,v\right)={u}^{\mathrm{\top }}v/\parallel u\parallel \parallel v\parallel$ represents cosine similarity between the object $u$ and $v$. Here $\tau$ represents a temperature parameter that scales the distance between $v,{v}^{+} \& {v}^{-}$.

#### Loss functions

The architecture of DCLGAN comprises of three distinct loss functions that guide model training. These loss functions are Adversarial loss, Identity loss and Patch NCE loss.

Patch noise contrastive estimation loss: ${G1}_{enc}$ and ${H}_{A}$ extract features from domain $\textcircled{A}$ and ${G2}_{enc}$ and ${H}_{B}$ extract features from domain $\textcircled{B}$. Layers $L$ from ${G1}_{enc}\textcircled{A}$ are sent to ${H}_{A}$, which embeds one image to a stack of features ${\left\{{z}_{l}\right\}}_{L}={\left\{{H}_{A}^{l}\left({G1}_{{\text{enc}}}^{l}\left(a\right)\right)\right\}}_{L}$, where ${G1}_{{\text{enc}}}^{l}$ denotes the output of $l$-th selected layers^14,15^ Considering that the image patches make up the stack of features extracted from the image, and each patch represents an individual feature present at locations $s$ in layers $L$, this can be expressed as a set $s\in \left\{1,\dots ,{S}_{l}\right\}$, here ${S}_{l}$ is the total number of spatial locations in every layer. Every query patch with its corresponding positive feature and its location is represented as ${z}_{l}^{s}\in {\mathbb{R}}^{{C}_{l}}$. All other features designated as negative in layers $l$ are expressed as ${z}_{l}^{S\backslash s}\in {\mathbb{R}}^{\left({S}_{l}-1\right)\times {C}_{l}}$, ${C}_{l}$ representing the total number of color channels in each layer. For the generated image $G1\left(a\right)$ belonging to domain $\textcircled{B}$, dual learning can be used to learn a different embedding of domain $\textcircled{B}$ to get another set of features $\left\{ {\hat{z}_{l} } \right\}_{L} = \left\{ {G2_{Y}^{l} \left( {G2_{{\text{enc }}}^{l} \left( {G1\left( a \right)} \right)} \right)} \right\}_{L}$.

**Figure 6:**
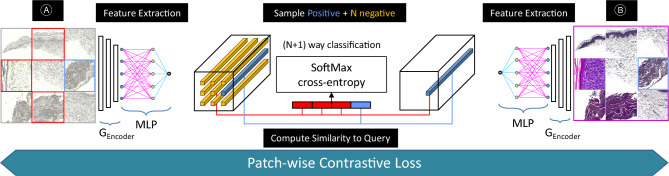
The Patch Noise Contrastive Estimation loss^15^ helps the generated image patch to look more like its real input shown in blue) while making it less like the other unrelated patches (shown in red).

Figure 6 explains the process of patch-wise contrastive loss that is applied to increase the similarity between real and generated patches. The patch-wise, multilayered Patch Noise Contrastive Estimation loss for mapping $G1:A\to B$ can be stated as:

$${\mathcal{L}}_{{PatchNCE_{A} ~}} \left( {G1,H_{A} ,H_{B} ,A} \right) = {\mathbb{E}}_{{a \sim A}} \mathop \sum \limits_{{l = 1}}^{L} \mathop \sum \limits_{{s = 1}}^{{S_{l} }} \ell \left( {\hat{z}_{l}^{s} ,z_{l}^{s} ,z_{l}^{{S\backslash s}} } \right)$$

For learning the reverse mapping patch NCE loss $G2:B\to A$, the loss function will be,

$${\mathcal{L}}_{{PatchNCE~_{B} }} \left( {G2,H_{A} ,H_{B} ,B} \right) = {\mathbb{E}}_{{b \sim B}} \mathop \sum \limits_{{l = 1}}^{L} \mathop \sum \limits_{{s = 1}}^{{S_{l} }} \ell \left( {\hat{z}_{l}^{s} ,z_{l}^{s} ,z_{l}^{{S\backslash s}} } \right)$$

${\left\{{z}_{l}\right\}}_{L}={\left\{{G2}_{B}^{l}\left({G2}_{{\text{enc}} \, }^{l}\left(b\right)\right)\right\}}_{L}$ and $\left\{ {\hat{z}_{l} } \right\}_{L} = \left\{ {G2_{A}^{l} \left( {G1_{{\text{enc }}}^{l} \left( {G2\left( b \right)} \right)} \right)} \right\}_{L}$ are different from $G1:A\to B$

Adversarial loss: Adversarial loss^26^ is used to guide the generator to generate realistic images with similar visual appearance to the ground truth images. There are two generators used for learning two inverse mappings across the domains which is why there are two different adversarial losses. The loss function for mapping $G1 : A \to B$ using discriminator ${D}_{B}$ is.

$$\begin{array}{l}{\mathcal{L}}_{GAN}\left(G1,{D}_{B},A,B\right) ={\mathbb{E}}_{b\sim B}\left[{\text{log}}{(D}_{B}\left(b\right))\right]+{\mathbb{E}}_{a\sim A}\left[{\text{log}}\left(1-{D}_{B}\left(G1\left(a\right)\right)\right]\right.\end{array}$$

Here $G1$ generates images $G1\left(a\right)$ that are similar in appearance to images of domain B and discriminator ${D}_{B}$ differentiates between generated images $G1\left(a\right)$ and real images of domain B. The Loss function for mapping $G2 : B \to A$ using discriminator ${D}_{A}$ is.

$$\begin{array}{l}{\mathcal{L}}_{GAN}\left(G2,{D}_{A},A,B\right) ={\mathbb{E}}_{a\sim A}\left[{\text{log}}\left({D}_{A}\left(a\right)\right)\right]+{\mathbb{E}}_{b\sim B}\left[{\text{log}}\left(1-{D}_{A}\left(G2\left(b\right)\right)\right]\right.\end{array}$$

Identity loss: The idea of identity loss^11^ is implemented to ensures that the generators do not make unreasonable variations while generating image patches. Including identity loss while learning model embeddings also helps to preserve color in generated images. Identity loss can be represented as

$$\begin{array}{l}{\mathcal{L}}_{\text{identity }}\left(G1,G2\right)={\mathbb{E}}_{a\sim A}\left[\parallel G2\left(a\right)-a{\parallel }_{1}\right] +{\mathbb{E}}_{b\sim B}\left[\parallel G1\left(b\right)-b{\parallel }_{1}\right]\end{array}$$

#### Objective

The objective of using DCLGAN is to generate realistic looking H&E-stained images from unstained tissue biopsy samples keeping correspondence between both histological domains unstained (A) and stained (B). This can be expressed using a collective loss function as

$$\begin{array}{cc}& \\ \mathcal{L}\left(G1,G2,{D}_{A},{D}_{B},{H}_{A},{H}_{B}\right) =& \begin{array}{c}{\lambda }_{GAN}\left({\mathcal{L}}_{GAN}\left(G1,{D}_{B},A,B\right)+{\mathcal{L}}_{GAN}\left(G2,{D}_{A},A,B\right)\right) \\ +\\ {\lambda }_{NCE} {\mathcal{L}}_{{\text{Patch NCE}}_{A} \, }\left(G1,{H}_{A},{H}_{B},A\right)+{\lambda }_{NCE}{\mathcal{L}}_{{\text{Patch NCE }}_{B}}\left(G2,{H}_{A},{H}_{B},B\right) \\ +\\ {\lambda }_{\text{idt }}{\mathcal{L}}_{\text{identity }}\left(G1,G2\right)\end{array}\end{array}$$

The relative weight values of various DCLGAN losses are ${\lambda }_{GAN}=1,{\lambda }_{NCE}=2$ and ${\lambda }_{idt}=1$.

### Merging patches at inference stage

**Figure 7:**
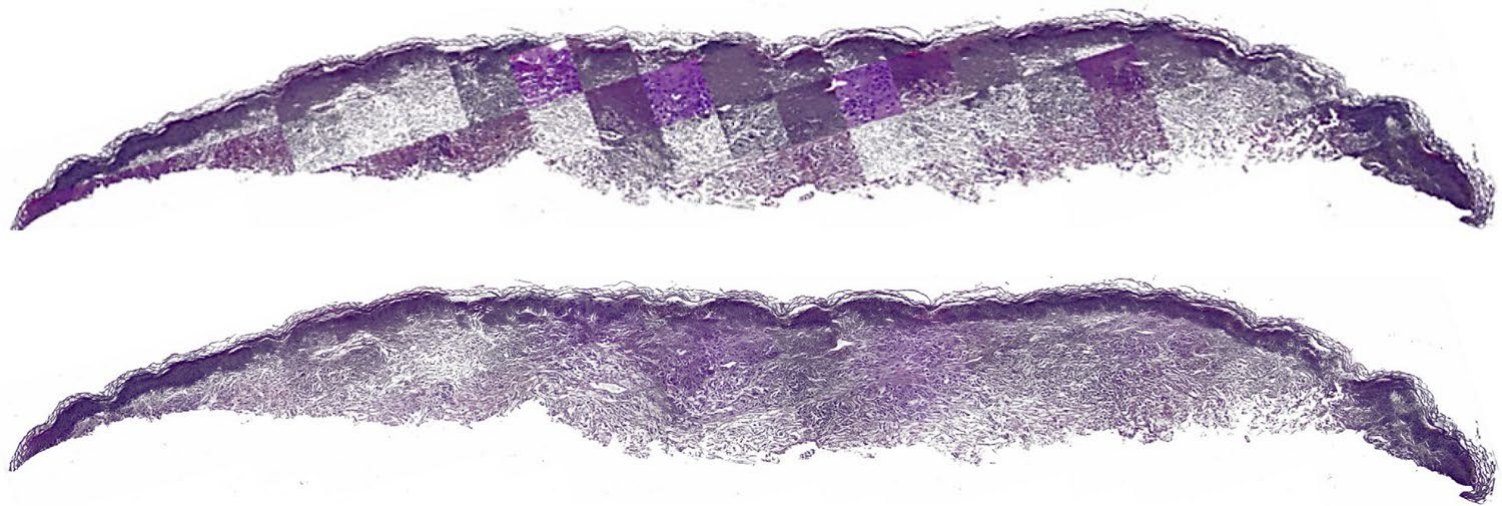
The top image demonstrates the patchy appearance of DCLGAN’s output with artifacts. In contrast, the bottom tissue image shows the result after overlapping and blending patches, resulting in a smooth seamless image without any artifacts.

Microscopic slides can be very large, ranging from tens of megapixels to gigapixels. Therefore, they cannot be directly inputted into the GAN models. Instead, they are fed to the models in smaller patches, resulting in virtual patches that can vary significantly in color and contrast, even for the same image. At the inference stage, the model processes each patch individually, creating artifacts at patch boundaries in the final composite image. To address this issue, a solution was devised to overlap patches by 50% in both horizontal and vertical directions, and alpha-blend the overlapping areas. A weight matrix assigned different weights to corners, edges, and center tiles to achieve seamless blending. The resulting image had smooth edges, with no visible artifacts or patchy appearance. Figure 7 illustrates the effectiveness of this mechanism, showing the virtually stained whole slide image before and after blending.

## Experimental setup and evaluation criteria

**Figure 8:**
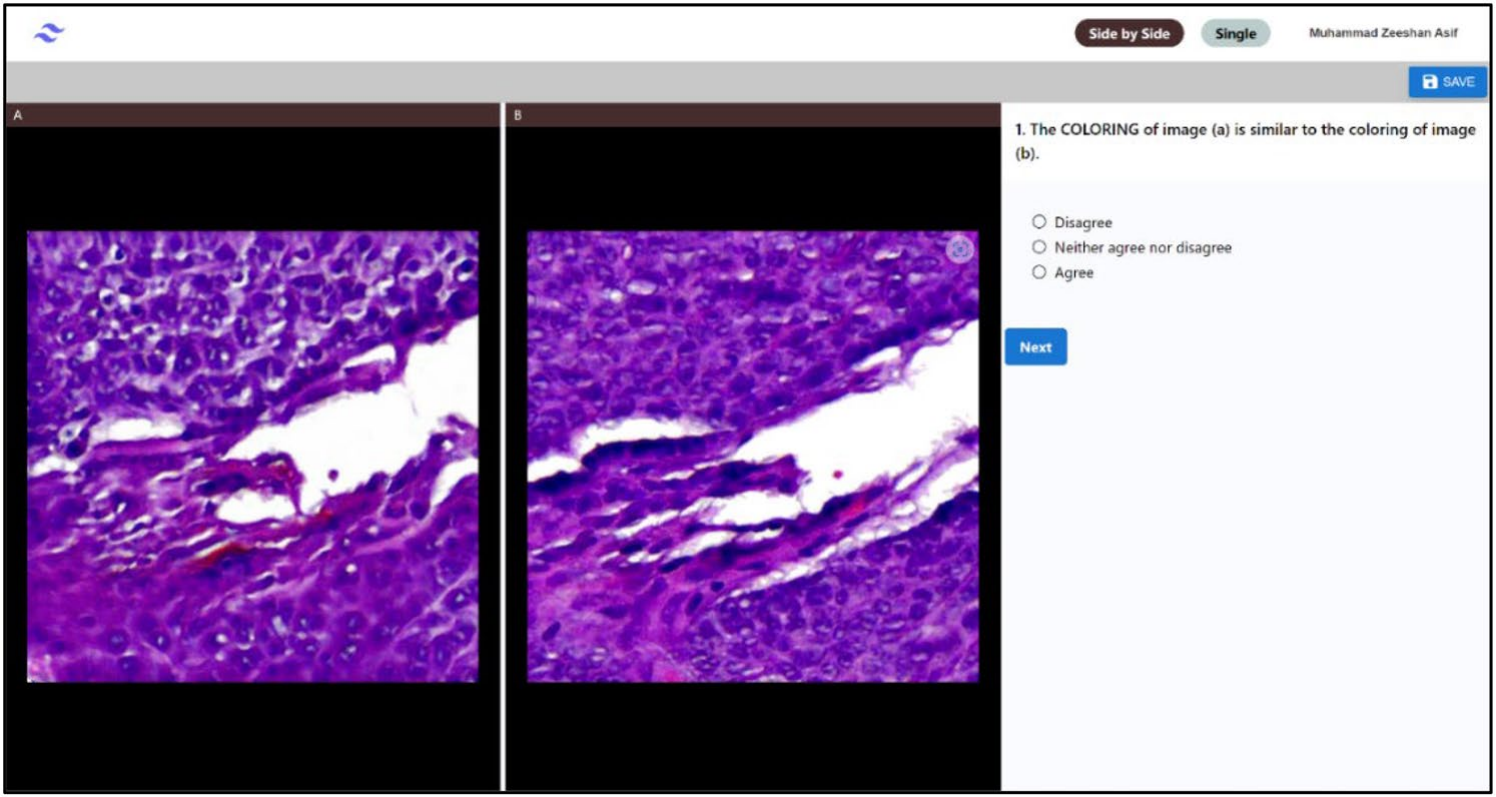
The web application interface for dermatopathologists to evaluate virtual and histologically stained tissues.

The models were trained on a desktop PC with Windows 11 Education as Operating System, Intel Core I7-8700k processor, 32 GB RAM and NVIDIA A6000 GPU with 48 GB VRAM. The higher VRAM of the GPU made it possible to train the model on relatively larger image patches. A web application (Fig. 8) was developed that allowed dermatopathologists to evaluate virtual and histologically stained tissues for various features in a blinded manner.

### Evaluation criteria

To validate the proposed virtual staining methodology, quantitative and qualitative assessments of virtually stained images were performed. The quantitative analysis was done using different matrices i.e., Fréchet Inception Distance and Kernel Inception distance^29,30^. The qualitative and visual evaluation was performed using dermatopathologists as graders. The image graders’ evaluations are further quantified to assess the overall similarity between original H&E stained and virtually stained patches. These matrices reflect the quality of the virtual staining models and how they resembled traditional histological stains.

#### Fréchet inception distance

Fréchet Inception Distance (FID)^29^ is used to compare the virtual stains to H&E stains. FID shows high correspondence with human perception and estimates the distribution of real and generated images in a network feature space, computing the divergence between them using Fréchet distance. Feature vectors for FID are extracted from a pretrained Inceptionv3 model. Lower values represent better results and higher correspondence between the two image distributions for FID.

#### Kernel inception distance

Kernel Inception distance (KID)^18^ is another metric used to assess the similarity between real and generated images. Similar to FID, it also uses a pre-trained Inceptionv3 model to extract features from both image distributions, but instead of using Fréchet distance, it finds the squared maximum mean discrepancy using a polynomial kernel. Like FID, lower values represent better results and higher correspondence between the given image distributions.

#### Evaluation by dermatopathologists

Numerical values from evaluation criteria can reflect on the quantified quality of the generated virtual stain, but these cannot replace assessment by pathologists. Therefore, three board-certified dermatopathologists with 35 years of collective experience evaluated virtual and histologically stained tissues to assess the similarity between images. Slides were evaluated for coloring, resolution, sharpness, contrast, brightness, uniform illumination, and the presence of artifacts. Visibility of melanocytes, keratinocytes, and inflammatory cells were also noted.

## Results

**Figure 9:**
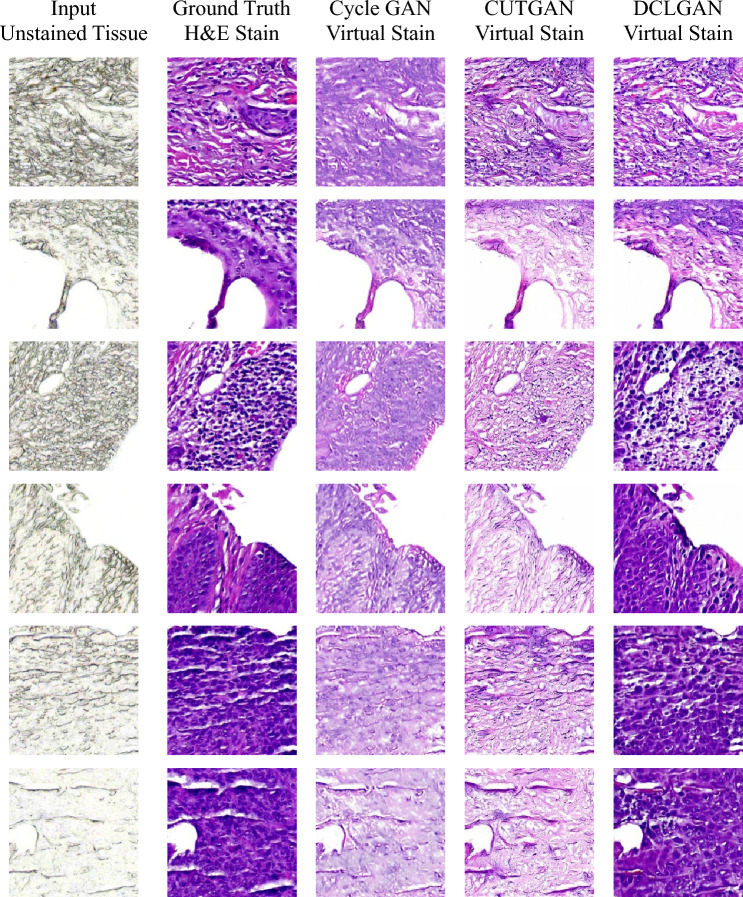
Comparative result of different virtual staining methods. The image shows the input unstained tissue, the ground truth H&E stain, and the virtual stains produced by Cycle GAN, CUTGAN, and proposed DCLGAN models.

The data was trained on three different GAN models Cycle GAN, CUTGAN, and DCLGAN. Figure 9 shows examples of how each of these models performed against the ground truth H&E stain.

The results indicate superior performance of DCLGAN model compared to the other two models. DCLGAN outperforms Cycle GAN and CUTGAN in terms of generating virtual stains as it is more detailed, contrasted, and similar to the ground truth H&E stain. The structures and textures in the tissues are more distinct and visible in the DCLGAN. The results generated by Cycle GAN are a purplish recolored image of the unstained tissue not including any additional information that required geometrical changes. Whereas the CUTGAN generated images are more blurry, faded, and inconsistent compared to DCLGAN’s result. Furthermore, for this research work supervised GAN models such as pix2pix etc., were not used. Supervised GAN models require paired data for good results. This is hard to obtain as tissues deform during staining. This deformity makes it difficult to do pixel level alignment of unstained and H&E-stained images needed by these models. Therefore, only DCLGAN that generated good results with tissue data was evaluated.

**Figure 10:**
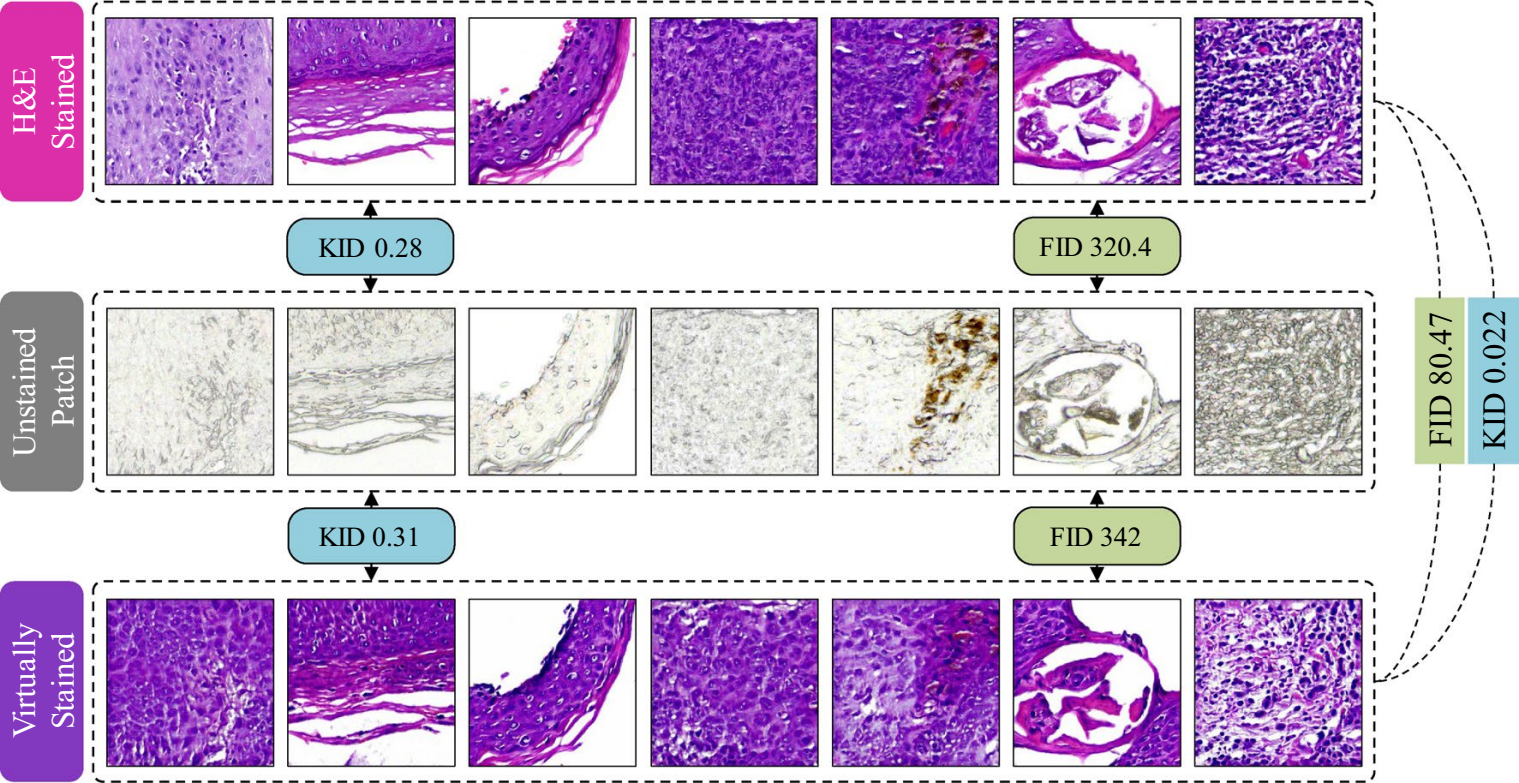
Unstained, H&E stained and virtually stained patches along with respective quantified average FID and KID scores.

FID and KID are used to evaluate real and generated image distributions using a pretrained Inceptionv3 model. Both metrics align well with human perception. Figure 10 visually shows the 3 sets of pairs for the calculation of FID and KID. To calculate FID, the images are resized to 299 × 299 pixels and covariance and mean values of 2048 features are computed to measure the FID score. Three sets of FID values are generated by comparing three image distributions, unstained images with H&E-stained images (320.4), unstained images with virtually stained images (342), and the H&E-stained images with virtually stained images (80.47). The comparison between H&E-stained and virtually stained images has a comparatively lower FID score of 80.47, indicating that the proposed model is able to produce realistic and similar images to the H&E-stained images. The higher FID scores between unstained and H&E stained (320.4) as well as unstained and virtually stained (342.01) indicate lower similarity between the two image distributions. However, the small difference of 6.3% in FID score between unstained and H&E stained and unstained and virtually stained can also be interpreted as that both datasets and their image distributions are similar in terms of features since both are approximately at the same distance from the unstained data.

Similarly, KID is computed using the maximum mean discrepancy. Lower KID values show greater similarity between the datasets. The mean KID score between H&E stained and virtually stained images is significantly lower (0.022) than the mean KID score between unstained and H&E stained (0.28) or unstained and virtually stained (0.31) images.

**Figure 11:**
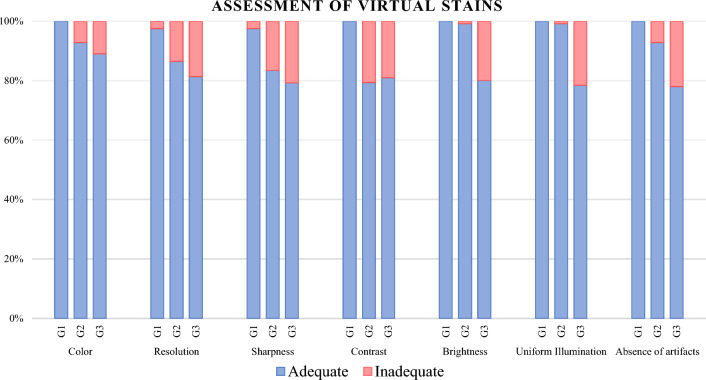
Grading of virtually stained single images for the following seven features: color, resolution, sharpness, contrast, brightness, uniformity of tissue illumination, and absence of artifacts, grading them as Adequate (blue) or Inadequate (pink).

Three dermatopathologists evaluated (1) single virtual patches and (2) side-by-side H&E and virtual patches. In (1) single virtual patches, graders were presented with 236 randomly selected image patches. Dermatopathologists (graders G1, G2, G3) evaluated the patches as Adequate or Inadequate across seven features (Table 1). Figure 11 depicts grader response to the provided quality assessment questions.

**Table 1:** Graders' assessment of single image virtually stained patches across seven features.

Feature	Color			Resolution			Sharpness			Contrast			Brightness			Uniform illumination			Absence of artifacts		
Grader	G1	G2	G3	G1	G2	G3	G1	G2	G3	G1	G2	G3	G1	G2	G3	G1	G2	G3	G1	G2	G3
Adequate	236	219	210	230	204	192	232	197	187	236	188	191	236	234	189	236	234	185	233	219	184
N %	100%	92.8%	89%	97.5%	86.4%	81.4%	98.3%	83.5%	79.2%	100%	79.7%	80.9%	100%	99.2%	80.1%	100%	99.2%	78.4%	98.7%	92.8%	78%
All grader s (3/3 agree)	193			163			154			149			188			184			167		
N %	81.8%			69.1%			65.3%			63.1%			79.7%			78.0%			70.8%

Table 1 shows the grading results of single image patches as either adequate or inadequate by the three graders across 7 categories. The results show an average percentage agreement of 72.5% across all features. All graders show the highest agreement on color (81.8%), followed by uniform brightness (79.7%) and illumination (78%). The lowest agreement is observed on sharpness (65.2%) and contrast (63.1%).

**Figure 12:**
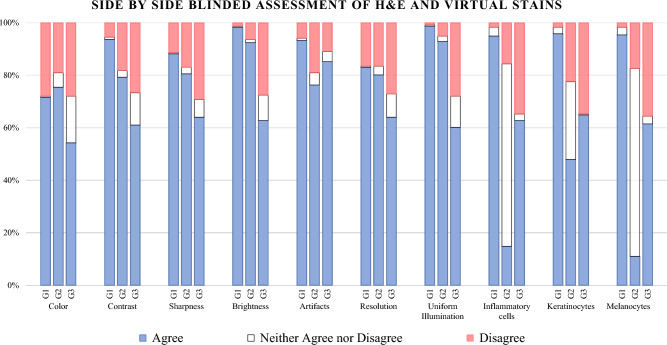
Comparison of assessment made by three graders on the similarity and quality of H&E stained and virtually stained images. The evaluators made a side-by-side comparison between images and rated agreement, disagreement, and neutrality across each pair for different features.

The second evaluation by the graders assessed the similarity between randomly selected matching pair of H&E and virtually stained H&E image patches. The evaluation was conducted in a blinded manner and the evaluators were not aware that out of the two given images, which one was histologically stained, and which one was model generated virtual H&E stain. Like the previous single image patch assessment, the pathologists compared the two stains based on the seven features of color, resolution, sharpness, contrast, brightness, uniform illumination, and absence of artifacts. In addition to these, three new features were included, the presence of melanocytes, keratinocytes, and inflammatory cells. Figure 12 highlights the scoring of the side-by-side images for the mentioned categories. Pathologists were given the following statement: the {FEATURE} of image (a) is similar to the {FEATURE} of image (b). Pathologists responded to the statement on a three-point scale labelled as: Agree, Neither Agree nor Disagree (N.A.D.A) and Disagree.

The comparison between H&E and virtual stains revealed that for the following features: color, resolution, sharpness, contrast, brightness, uniform illumination, absence of artifacts, the percent agreement between three graders ranged from 54.3 to 98.3% with an average agreement of 78.8%. When determining whether all 3 graders AGREE that pairs of image patches were similar, the percentage agreement ranged from 44.1% (Color) to 65.3% (absence of artifacts).

The most variation in grader response was the presence of inflammatory cells, keratinocytes, and melanocytes. The average value of AGREE across these three cell types varied from 24.58% (G2) to 95.34% (G1) (Table 2). In cases where melanocytes, keratinocytes, and inflammatory cells were not visible, pathologists responded to the statement as "neither agree nor disagree". Graders noted the need for further testing of the model with whole-slide images and the diagnosis of dermatologic tumors and conditions.

**Table 2:** Side-by-side blinded comparison between histologically stained H&E and virtually generated H&E stain.

Feature	Color			Resolution			Sharpness			Contrast			Brightness		
Grader	G1	G2	G3	G1	G2	G3	G1	G2	G3	G1	G2	G3	G1	G2	G3
Agree	169	178	128	196	189	151	208	190	151	221	187	144	232	218	148
N %	71.6%	75.4%	54.2%	83.1%	80.1%	64%	88.1%	80.5%	64%	93.6%	79.2%	61%	98.3%	92.4%	62.7%
N.A. D.A	1	13	42	1	8	21	1	6	16	2	6	29	1	3	23
N %	0.4%	5.5%	17.8%	0.4%	3.4%	8.9%	0.4%	2.5%	6.8%	0.8%	2.5%	12.3%	0.4%	1.3%	9.7%
Disagree	66	45	66	39	39	64	27	40	69	13	43	63	3	15	65
N %	28%	19.1%	28%	16.5%	16.5%	27.1%	11.4%	16.9%	29.2%	5.5%	18.2%	26.7%	1.3%	6.4%	27.5%
All graders (3/3 agree)	104			125			133			131			145		
N %	44.1%			53%			56.4%			55.5%			61.4%		

Feature	Uniform illumination			Artifacts			Melanocytes			Keratinocytes			Inflammatory cells		
Grader	G1	G2	G3	G1	G2	G3	G1	G2	G3	G1	G2	G3	G1	G2	G3
Agree	233	219	142	220	180	201	225	26	145	226	113	153	224	35	148
N %	98.7%	92.8%	60.2%	93.2%	76.3%	85.2%	95.3%	11%	61.4%	95.8%	47.9%	64.8%	94.9%	14.8%	62.7%
N.A. D.A	1	5	28	2	11	9	7	169	7	6	70	1	8	164	6
N %	0.4%	2.1%	11.9%	0.8%	4.7%	3.8%	3%	71.6%	3%	2.5%	29.7%	0.4%	3.4%	69.5%	2.5%
Disagree	2	12	66	14	45	26	4	41	84	4	53	82	4	37	82
N %	0.8%	5.1%	28%	5.9%	19.1%	11%	1.7%	17.4%	35.6%	1.7%	22.5%	34.7%	1.7%	15.7%	34.7%
All graders (3/3 agree)	137			154			20			94			29		
N %	58.1%			65.3%			8.5%			39.8%			12.3%		

Grading pathologists rated 10 features (color, resolution, sharpness, contrast, brightness, uniform Illumination, absence of artifacts, presence of melanocytes, keratinocytes, and inflammatory cells) on three-point Likert scale as agree, neither agree nor disagree (N.A.D.A) and disagree.

## Limitations and future work

This study has some limitations that should be acknowledged and addressing them is planned in future work. First, our virtual staining method based on DCLGAN relies on data primarily obtained from a single source (NIDI skin). This limits the generalizability of the results across different laboratory settings, as variations in staining protocols among labs result in vastly different looking stains. This can significantly impact the model’s performance. To address this limitation, we plan to collect more additional data from different sources to enhance the diversity and application of the virtual staining models. Second, obtaining aligned pairs of unstained and stained image data poses a significant challenge. This is due to tissue deformation caused during staining, making it challenging to register unstained and stained tissue images accurately. To overcome this challenge, we aim to develop improved methods for registering and aligning images, possibly exploring techniques such as elastic nonlinear deformation based methods. Third, the proposed methodology, while an improvement over manual staining, takes several minutes to process a moderately sized unstained slide. Although relatively efficient, To further reduce processing time, we intend to incorporate segmentation models to exclude background areas that do not contribute useful information for virtual staining.

In this paper, we have proposed a novel method for virtual staining of unstained skin tissue samples using deep convolutional generative adversarial networks (DCLGANs). Our method can generate realistic and diverse H&E stained images that preserve the fine details and structures of the original tissue, while avoiding the drawbacks of the traditional staining process. However, our study also has some limitations and challenges that open up opportunities for future work. Some of the possible directions for future work are:

- Diversification of Data Sources: Future work should prioritize the collection of data from various sources to enhance the diversity of virtual staining models. This will help in developing more robust models capable of adapting to variations in staining protocols across different settings.
- Improved Paired Data Acquisition: Developing and using better methods for registering and aligning unstained and stained images, possibly using elastic nonlinear deformation based methods.
- Exploration of Large Vision Models (LVMs): Future work should explore the use of image-to-image translation GAN models based on large vision models (LVMs). Investigating improved quality in virtual staining tasks.
- Optimization of Processing Time: Efforts should be directed towards reducing the time consumption of the virtual staining methodology. Implementing segmentation models to discard unrelated background and using more efficient model architectures for this purpose.
- Expansion to Other Tissues: The methodology’s applicability should be extended to virtual staining of other types human tissues, such as Colon, Breast, and Lungs.

## Conclusion

Deep learning models have the potential to revolutionize the processing and analysis of histology slides. In this study, we proposed a novel dual contrastive learning generative adversarial network for image-to-image translation of unstained skin tissue images into virtually stained H&E images. The proposed methodology takes an average of seven minutes to process a 25,000 × 25,000 unstained slide into a virtually stained whole slide image. Fréchet Inception Distance and Kernel Inception distance were used to quantify the quality of the virtual staining models, which reflected how closely virtual H&E stains matched with histological H&E stains. Furthermore, three experienced dermatopathologists positively evaluated the quality of the virtual stain across seven features. This study demonstrates the potential use of GAN models in virtual staining, which could offer advantages in reducing time and the environmental impact associated with traditional staining.

## Data Availability

We have made the data publicly available at our research group website https://biomisa.org/index.php/downloads/ with the title Skin Histology Data. For further details about data, please contact corresponding author at usmakram@gmail.com.
